# Dataset on the cost estimation for spent filter backwash water (SFBW) treatment

**DOI:** 10.1016/j.dib.2017.10.040

**Published:** 2017-10-24

**Authors:** Afshin Ebrahimi, Mokhtar Mahdavi, Meghdad Pirsaheb, Fariborz Alimohammadi, Amir Hossein Mahvi

**Affiliations:** a Department of Environmental Health Engineering, Environment Research Center, Research Institute for Primordial Prevention of Non Communicable Disease, Isfahan University of Medical Sciences, Isfahan, Iran; b Environmental Health Engineering Department, Saveh University of Medical Sciences, Social Determinants of Health Research Center, Saveh, Iran; c Department of Environmental Health Engineering, Kermanshah Health Research Center (KHRC), Kermanshah University of Medical Sciences, Kermanshah, Iran; d Experienced expert of Isfahan Water and Wastewater Company, Master of Sciences in Environmental Health Engineering, Iran; e Center for Solid Waste Research, Institute for Environmental Research, Tehran University of Medical , Tehran, Iran

**Keywords:** Spent filter backwash water, Water treatment, Coat estimation, Water reuse

## Abstract

The dataset presented in this article are related to the research article entitled “Hybrid coagulation-UF processes for spent filter backwash water treatment: a comparison studies for PAFCl and FeCl_3_ as a pre-treatment” (Ebrahimi et al., 2017) [1]. This article reports the cost estimation for treating produced spent filter backwash water (SFBW) during water treatment in Isfahan- Iran by various methods including primary sedimentation, coagulation & flocculation, second clarification, ultra filtration (UF) and recirculation of settled SFBW to water treatment plant (WTP) entrance. Coagulation conducted by PAFCl and FeCl_3_ as pre polymerized and traditional coagulants. Cost estimation showed that contrary to expectations, the recirculation of settled SFBW to WTP entrance is more expensive than other method and it costs about $ 37,814,817.6. Versus the cheapest option related to separate primary sedimentation, coagulation & flocculation in WTP. This option cost about $ 4,757,200 and $ 950,213 when FeCl3 and PAFCl used as coagulant, respectively.

**Specifications Table**

**Table:** 

Subject area	Environmental engineering
More specific subject area	Water reuse
Type of data	Table
How data was acquired	– Experimental results attained from pilot plant that include primary sedimentation, coagulation & flocculation, ultra filter and recirculation of settled SFBW to WTP entrance. – Cost estimation for full scale treatment for SFBW. – Cost estimation for different method that proposed for SFBW treatment including: mixing of settled SFBW with raw water entered to WTP, Separate primary sedimentation, coagulation & flocculation in WTP, Separate primary sedimentation, coagulation & flocculation and secondary sedimentation in WTP and Separate primary sedimentation, coagulation & flocculation, secondary sedimentation and UF process in WTP
Data format	Raw and analysed
Experimental factors	Application of sedimentation, coagulation & flocculation, UF process and return of SFBW to WTP for SFBW treatment Cost estimation for each process according to dimension, chemical consumption and necessary equipment.
Experimental features	Determination of cost and feasibility of selected method for SFBW treatment
Data source location	Isfahan's WTP in Iran
Data accessibility	Some data are within this article and some presented in published article. Of course published data was presented in this article but with reference number and citation.

**Value of the data**

• The data presents the suitable method among recirculation of settled SFBW to WTP entrance, coagulation & flocculation, and ultra filtration process for SPBW treatment
• Cost estimation for SFBW reuse by mentioned methods at full scale.
• Effect of cost estimation on process selection and Vice versa.

## 1 Data

The dataset of this article provides information on the cost estimation of SFBW treatment by various methods, including recirculation of settled SFBW to WTP entrance, coagulation & flocculation, and ultra filtration process. Coagulation conducted with two different coagulant including PAFCl and FeCl_3_. Table 1, Table 2, Table 3 show the amount of coagulants consumption (according to optimum dose of coagulants) and cost estimation for all process that used for SFBW treatment at full scale (Q=24,000 m^3^/d). Also all dimension, instrument, chemical matter and required parameters for water treatment plant were estimated and used for estimation.

**Table 1:** The amount and cost of coagulant that is need for treating SFBW at full scale.

**Parameters**	**Full scale**^c^	
	FeCl_3_	PAFCl
Optimum dose^a^ (mg/L)	40 and 30	15 and 10
Annual consumption (kg)	302,400	108,000
Consumption during design period (kg)	7,560,000	2,700,000
The annual cost (USD)^b^	181,440	29,160
Total cost during design period (USD)	4,536,000	729,000

a In this study the optimum doses of FeCl_3_ and PAFCl for autumn and winter was 40 and 15 mg/L, respectively and for spring and summer were 30 and 10 mg/L, respectively. So in this section cost data related to summation of two different amounts of doses during seasons.

b The average value cost of buying in global market for FeCl_3_ in 2016 was about 600 USD per ton and for PAFCl was 270 USD per ton.

c Design period for full scale was 25 years that operated daily with 24,000 m^3^/d entrance, but for pilot scale design period was 4 years and operated 12 h in day with 10 l/h inflow.

**Table 2:** Cost estimation for treating SFBW with primary sedimentation, coagulation & flocculation, secondary sedimentation and UF process i n a full scale (design period was 25 years and Q=24,000 m^3^/d) [4].

Units and processes	Section	Dimension or equipment	Cost per USD (US$)
Primary sedimentation	Civil construction	Reinforced concrete, 2 rectangular basin, L=45 m, W=9 m, H=3.8 m, t=2.5 h	70,714.3
	Electromechanical instrument	2 mobile bridge for sludge collection, 3 pumps and supplementary instrument	29,714.2
	Repair and reconstruction^a^	All mechanical instrument during design period	27,428
	Energy consumption^b^	All mechanical instrument used in primary sedimentation	11,142.8
Coagulation and flocculation	Civil construction	Reinforced concrete, for square coagulation basin L=1.9 m, W=1.9 m, H=2.75 m, t=30 S.	24,857.1
		for flocculation basin L=13 m, W=9 m, H=5.3 m, t=30 min.	
	Electromechanical instrument	Coagulation: 2 mixer with15 kw/h, gear box, shaft and supplementary instrument.	3485.7
		Flocculation: 3 mixers with 1 kw/h, gear box, bridge, shaft and supplementary instrument.	
	Repair and reconstruction	All mechanical instrument during design period	24,000
	Energy consumption	All mechanical instrument used in coagulation and flocculation	29,781.4
Secondary sedimentation	Civil construction	Reinforced concrete, 2 rectangular basin, L=50 m, W=10 m, H=4.5 m, t=4 h	86,571.42
	Electromechanical instrument	4 mobile bridge for sludge collection, 3 pumps and supplementary instrument	29,714.2
	Repair and reconstruction^a^	All mechanical instrument during design period	27,428
	Energy consumption^b^	All mechanical instrument used in secondary sedimentation	11,142.8
FeCl_3_ requirement during 25 year operation	During coagulation	Optimum dose of FeCl_3_ in this study for autumn and winter was 40 mg/L and for spring and summer was 30 mg/L.	4,536,000
PAFCl requirement during 25 year operation	During coagulation	Optimum dose of PAFCl in this study for autumn and winter was 15 mg/L and for spring and summer was 10 mg/L.	729,000
UF	UF process	500 module of PES UF, size of each modules was 8 in. ×40 in.	571,428
	Electromechanical instrument and Energy consumption^b^	2 feed pump, 2 backwash pump	266,857
	Repair and reconstruction a	All UF module and mechanical instrument during design period	2,293,428
	Chemical cleaning	Annual UF cleaning by NaOH and Citric acid during design period	950
staffs and employee	laborer, electromechanical expert, water operator and guard	Total staffs were 6 people, 15% increase for salary wage per year during 25 years.	428,571
Total cost for treatment by FeCl_3_ and UF with 30% increment as a safety factor	–	–	11,015,178
Total cost for treatment by PAFCl and UF with 30% increment as a safety factor	–	–	6,066,078

a Consumable instrument was replaced in 5 years interval over 25 years with an annual profit increase of 15%.

b Energy consumption for water and wastewater treatment plant in Isfahan is under agriculture industry. Power consumption Prices during 19 p.m. to 23 p.m. was 0.01257 USD, during 23 p.m. to 7 a.m. was 0.002 USD and during 7 a.m. to 19 p.m. was 0.00628 USD.

**Table 3:** Cost for SFBW treatment with different methods and process.

Method of treatment^*^	**Coagulant**	**Cost per USD (US$)**
Mixing of settled SFBW with raw water entered to WTP	PACl	37,814,817.6
		
Separate primary sedimentation, coagulation & flocculation in WTP	FeCl_3_	4,757,200
	PAFCl	950,213
		
Separate primary sedimentation, coagulation & flocculation and secondary sedimentation in WTP	FeCl_3_	4,912,000
	PAFCl	1,105,000
		
Separate primary sedimentation, coagulation & flocculation, secondary sedimentation and UF process in WTP	FeCl_3_	11,015,000
	PAFCl	6,066,000

## 2 Experimental design, materials and methods

### 2.1 Quantity of raw SFBW

Coagulation, flocculation, sedimentation and rapid sand filtration processes are main section of Isfahan water treatment plant that treats 12 m^3^/s of water. There are 48 filter units in this plant and PACl used as coagulant. During backwashing of each filter, some 500 m^3^ of wastewater was generated. Considering 48 filter with 24 h cleaning interval it accounts for about 2.25% of the raw water entering to the plant. So, during water treatment process approximately 24,000 m^3^/d of SFBW is generated.

### 2.2 Experimental procedure

In our previous study, continues processes including primary sedimentation, coagulation, flocculation, secondary sedimentation and UF were used for the SFBW treatment. Inflow of all sections of the pilot except UF membrane was 10 l/h. Hydraulic retention time (HRT) for mentioning sections, except UF membrane was 60, 6, 48 and 192 min. Optimum pH for coagulation with PAFCl and FeCl_3_ was 8.3. Also, optimum doses of PAFCl and FeCl_3_ were 10 mg/L and 30 mg/L for spring and summer and 15 mg/L and 40 mg/L for autumn and winter seasons. Mixing speed at rapid mixer basin was 80 rpm. Mixing speed at flocculation tanks was 48 rpm. The UF module was operated in a dead-end mode with constant filtration about 8 L m^−2^ h^–^^1^ at a trans-membrane pressure of 300 Pa. It was operated in a cycle of 60 min filtration and 1 min backwashing with permeate in the reverse direction. At the end the recirculation of settled SFBW to WTP entrance and mixing with raw water was investigated according to its effects on coagulation usage at WTP in full scale situation [1], [2], [3], [4]. All dimensions for full scale treatment were designed and by considering civil construction materials, chemical consumption, equipments and other important parameters cost estimation was done.

The importance of proper treatment processes for SFBW is that in case there are some concentrations of pollutants being accumulated in the SFBW they will be removed to much lower concentrations with lower costs than advanced water treatment processes [5], [6], [7].
