# Supplementary figures and images for: Dataset on the cost estimation for spent filter backwash water (SFBW) treatment
Source: Data Brief. 2017 Oct 24;15:1043–7. doi: 10.1016/j.dib.2017.10.040 (PMC5695916; doi:10.1016/j.dib.2017.10.040)

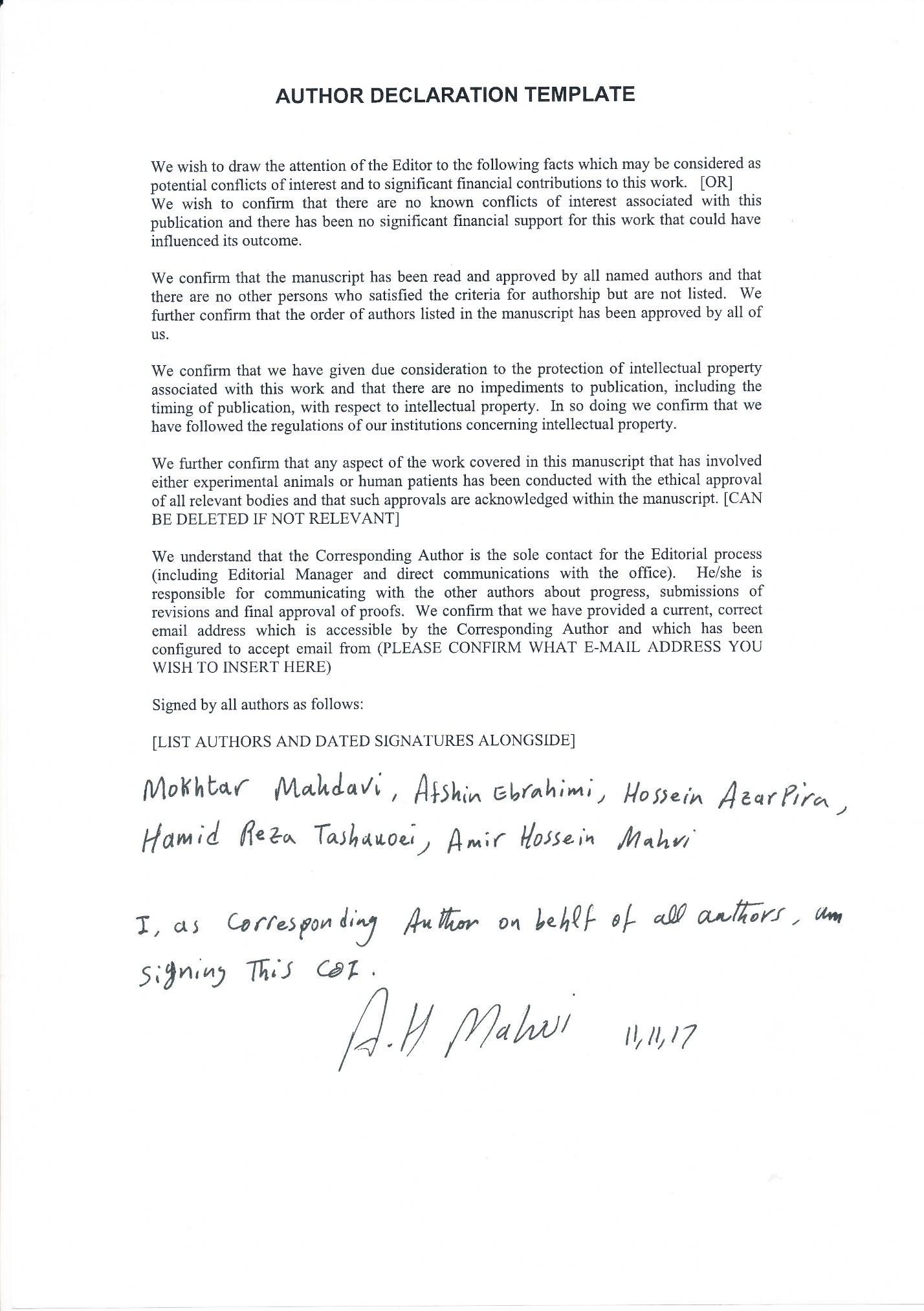

Supplement: Supplementary file 1 — Supplementary material [file mmc1.docx]
